# Occurrence, dietary exposure, and health risk estimation of polycyclic aromatic hydrocarbons in grilled and fried meats in Shandong of China

**DOI:** 10.1002/fsn3.843

**Published:** 2018-10-16

**Authors:** Dafeng Jiang, Guoling Wang, Linlin Li, Xiaolin Wang, Wei Li, Xia Li, Lijun Shao, Fenghua Li

**Affiliations:** ^1^ Department of Physical and Chemical Testing Shandong Center for Food Safety Risk Assessment Shandong Center for Disease Control and Prevention Jinan China; ^2^ Shandong Institute for Product Quality Inspection Jinan China; ^3^ Department of Chemistry Liaocheng University Liaocheng China

**Keywords:** food safety, grilled and fried meat, polycyclic aromatic hydrocarbons, quantitative analysis, risk assessment

## Abstract

There is a lack of information regarding the quantitative determination and health risk assessment of polycyclic aromatic hydrocarbons (PAHs) in grilled and fried meat products in Shandong Province of China. The aim of this work was firstly to detect the contamination levels of 15 PAHs in 52 grilled and fried meats consumed by the population of Shandong Province, China. In brief, concentrations of the sum of 15 PAHs in individual samples were ranged from 8.23 to 341 μg/kg with a mean contamination level of 63.3 μg/kg. Moreover, the factors for the formation of PAHs in these samples have been identified and analyzed. One grilled meat sample exceeded the maximum limits of 2 and 12 μg/kg set for BaP and PAH4 by the European Union. For a further step, the mean dietary exposures for total PAHs from grilled and fried meat products were estimated to be 120 and 74.8 ng/kg bw/day, respectively. Finally, the health risk estimation was performed using the incremental lifetime cancer risk (ILCR) approach. The obtained values of four groups were all lower than 10‐4, indicating a slight potential carcinogenic risk of consumer health. This study was the first attempt to provide baseline information of potential health risk of dietary exposure of PAH‐containing grilled and fried meats, which could be useful for health management of the local consumers.

## 1 INTRODUCTION

Polycyclic aromatic hydrocarbons (PAHs), a group of highly hydrophobic and organic compounds consisting of two or more fused aromatic rings, are ubiquitous in environment (Purcaro, Moret, & Conte, 2013; Singh, Varshney, & Agarwal, 2016; Xia et al., 2010). PAHs are harmful to human health, and a number of them are carcinogenic, mutagenic, and genotoxic (Cai, Lv, Zhang, & Zhang, 2012; Martorell et al., 2010). Consequently, 16 PAHs were selected by the United State Environmental Protection Agency (USEPA) as priority pollutants based on their occurrence and relative carcinogenicity. The European Food Safety Authority (EFSA) also officially established a list of “15 + 1” European Union (EU) priority PAHs, which is distinguished from the 16 USEPA PAHs. Among these regulated PAHs, benzo(a)pyrene (BaP), classified as Group 1 (carcinogenic to humans), is the most investigated compound due to its proved carcinogenic activity (Alomirah et al., 2010;  Moret, Purcaro, & Conte, 2005). However, the EFSA found that BaP is not a sufficient indicator for PAH occurrence in food and suggested that the sum of benzo[a]anthracene (BaA), chrysene (Chr), benzo[b]fluoranthene (BbF), and BaP (PAH4), as well as the sum of BaA, Chr, BbF, BaP, benzo[k]fluoranthene (BkF), benzo[g,h,i]perylene (BghiP), dibenz[a,h]anthracene (DahA), and indeno[1,2,3‐cd]pyrene (IcdP) (PAH8), is the most suitable criterion (Alomirah et al., 2011; Li, Wu, Wang, & Akoh, 2016; Purcaro et al., 2013; Rozentale et al., 2015). Consequently, the EU reported that the maximum levels (MLs) for BaP and PAH4 in smoked meat products were 2 and 12 μg/kg, respectively (Commission Regulation (EC) No 1881/2006 amended by Commission Regulation (EU) No 835/2011).

Human beings can be easily suffered from these compounds via a variety of pathways. In particular, dietary intake of food is the major exposure route of PAHs for nonsmoking and nonoccupationally exposed populations (Domingo, 2014; Purcaro et al., 2013; Singh et al., 2016). The sources of PAH in food can come from food processing and preparation, cooking procedures, environmental contamination, and direct contact with nonfood grade mineral oil and contaminated package material (Purcaro et al., 2013). Generally, the amount of PAHs generated during the thermal food processing might cause by many parameters, such as temperature, duration of the treatment, distance from the source of heating, fat content, oxygen accessibility, and the type of combustible used (Akpambang et al., 2009; Essumang, Dodoo, & Adjei, 2013; Lee et al., 2016; Oz & Yuzer, 2016).

With the rapid economic growth and food structure change, meat and meat products have become daily food for most Chinese consumers (He, Yang, Xia, Zhao, & Yang, 2016). In particular, grilled and fried meat products are becoming increasingly popular in both homes and restaurants due to their well flavor and high nutritional values. Shandong, a coastal province in east China (34.61–37.91°N and 115.08–122.41°E), is an important industrial region and one of the top manufacturing provinces in China (Chai et al., 2017). Nearly 100 million people live in this region, where grilled and fired meat products represent a significant part of the daily diet. The aim of the present study was firstly to perform a PAH contamination survey on grilled and fried meat samples from the Shandong market of China. The second objective was to identify the major sources of PAHs in these samples. Finally, the dietary exposure and health risk estimation with the consumption of these foodstuffs were estimated.

## 2 MATERIALS AND METHODS

### 2.1 Chemicals and reagents

A certified solution of 16 USEPA priority PAHs with a concentration of 0.2 mg/ml for each, containing naphthalene (Nap), acenaphthylene (Acy), acenaphthene (Ace), fluorene (Fle), phenanthrene (Phe), anthracene (Ant), fluoranthene (Flu), pyrene (Pyr), benz(a)anthracene (BaA), chrysene (Chr), benzo(b)fluoranthene (BbF), benzo(k)fluoranthene (BkF), benzo(a)pyrene (BaP), dibenz(a,h)anthracene (DahA), indeno(1,2,3‐cd)pyrene (IcdP), and benzo(g,h,i)perylene (BghiP), was obtained from AccuStandard (New Haven, USA). In the present study, all solvents and reagents were of HPLC grade and analytical grade, respectively. Acetonitrile, methanol, acetone, n‐hexane, and dichloromethane were all obtained from Merck (Darmstadt, Germany). Ultrapure water was produced by a Milli‐Q purification water system (Millipore Co., USA).

### 2.2 Standard solutions and calibration curve

A series of working solutions, containing each PAH at concentration of 0.5, 1.0, 2.5, 5.0, 10, 25, and 50 ng/ml, were prepared by suitable dilution of the stock solutions with acetonitrile. The obtained solutions were stored at 4°C and renewed weekly.

### 2.3 Sampling and sample pretreatment

In the present study, 52 representative samples of various grilled (23) and fried (29) meat samples were purchased from the main retail outlets and local markets in 17 cities of Shandong province, China. The samples were collected during June to September in the year 2015. Each sample was homogenized and stored at −20°C until analyzed. If the samples contained nonedible parts, it should be removed firstly. It should also be pointed out that no further cooking procedure of the samples was done before analysis.

Approximately 20 g of the homogenized sample was weighed, and 100 ml of petroleum ether was added. Then, the sample was shaken for 30 s, ultrasonicated for 20 min, and centrifuged at 4500 *g* for 5 min. After the supernatant of the extracts was decanted, this procedure was repeated two more times. Finally, the combined extracts were rotary evaporated at 35°C to eliminate the solvents, and the residue was reconstituted in 5 ml of acetonitrile–acetone (v/v = 1:1) solution for further cleanup.

The cleanup procedures of the samples were performed based on the method described in our published study (Jiang et al., 2015). In brief, the extracts were cleanup on two sets of SPE columns, which were Oasis HLB column (WAT106202, 6 cc/200 mg) and Sep‐Pak Florisil (WAT043390, 6 cc/1 g) column, respectively. Thereafter, the final eluate was collected in a 10‐ml glass tube vial, evaporated under nitrogen stream at 35°C, and then reconstituted with 1 ml of acetonitrile–toluene (v/v = 9:1) before injection into the HPLC system.

### 2.4 HPLC analysis

A high‐performance liquid chromatography system (waters, made in Singapore) combined with a fluorescence detector was used for PAHs determination. Separation of analytes was carried out on a Waters PAH C18 analytical column (4.6 × 250 mm, 5 μm) maintained at 35°C. A flow rate of 1.0 ml/min was selected, and the injection volume was 10.0 μl. The gradient elution procedure, consisted of acetonitrile (A) and water (B), was performed as follows: 50% A (0–5 min), 50% A–100% A (5–20 min), 100% A (20–26 min), 100% A–50% A (26–27 min), and 50% A (27–32 min), giving a total time of 32 min. Fluorescence detection was performed by switching the emission and excitation wavelength in time (Table 1).

**Table 1:** The wavelength program for the fluorescence detection of individual PAHs

Time (min)	λ_ex_ (nm)	λ_em_(nm)	PAHs detected
7.7	224	330	Nap
11.0	270	323	Ace, Fle,
12.5	250	390	Phe, Ant
14.6	250	420	Flu, Pyr
18.0	270	385	BaA, Chr
20.0	280	410	BbF, BkF, BaP, DahA, BghiP
24.6	305	480	IcdP

### 2.5 Method validation and quality control

The data acquisition and analysis were processed with the Empower 2 software. Quantification was performed by an external standard method. The method was validated for linearity, accuracy, precision, limit of detection (LOD), and quantification (LOQ). Linearity was evaluated by constructing a standard curve for each PAH in the range of 0.5–50 ng/ml. Results demonstrated that all standard curves displayed good linearity with the correlation coefficients (*r* ^*2*^) higher than 0.992, as displayed in Table 2. Accuracy and intraday precision were determined by recovery experiments, which were conducted by spiking the samples with PAHs standards at levels of 2, 10, and 50 μg/kg (*n* = 5), respectively. Inter‐day precision was performed by repeating this procedure on three consecutive working days. The recoveries ranged from 71.3% to 123% (Table 2), indicating good accuracy of the method. The intra‐ and inter‐day precision was expressed as relative standard deviation (RSD). As shown in Table 2, the intra‐RSD and inter‐RSD values were in the range of 3.5%–13.8% and 4.9%–15.6%, respectively. The LODs and LOQs were calculated based on the analyte concentration giving a signal‐to‐noise of at least threefold (S/N > 3) and 10‐fold (S/N > 10), respectively. The LODs and LOQs for 15 PAHs were in the range of 0.06–0.30 μg/kg and 0.20–1.00 μg/kg, respectively.

**Table 2:** Validation parameters for the proposed method for the determination of target PAHs

PAHs	*r* ^*2*^	LOQ (μg/kg)	Level 1 (2 μg/kg)			Level 2 (10 μg/kg)			Level 3 (50 μg/kg)		
			Recovery (%)	Intra‐RSD (%)	Inter‐RSD (%)	Recovery (%)	Intra‐RSD (%)	Inter‐RSD (%)	Recovery (%)	Intra‐RSD (%)	Inter‐RSD (%)
Nap	0.9993	0.30	78.9	7.3	6.6	77.6	8.7	7.9	81.9	4.6	5.3
Ace	0.9985	0.30	80.7	5.9	8.4	76.1	8.2	6.8	83.2	7.9	8.1
Fle	0.9999	0.30	81.2	8.4	9.8	93.1	5.8	5.3	96.0	4.7	6.0
Phe	0.9921	1.00	107	13.8	15.6	95.9	7.8	9.4	103	8.1	7.2
Ant	0.9993	0.20	89.1	8.8	9.1	93.3	6.1	6.7	99.4	5.4	5.9
Flu	0.9937	0.30	115	6.7	8.1	121	4.3	5.5	123	3.5	5.3
Pyr	0.9996	0.30	111	10.8	9.3	116	10.1	9.2	107	6.9	7.3
BaA	0.9998	0.20	101	7.2	7.8	97.5	6.7	7.1	94.2	4.9	5.7
Chr	0.9996	0.20	89.9	5.9	8.4	83.7	4.0	5.6	91.8	3.8	4.9
BbF	0.9992	0.20	106	8.3	10.5	95.4	4.8	5.2	109	4.9	6.3
BkF	0.9995	0.20	83.3	6.0	6.5	80.5	7.1	7.8	85.7	5.5	7.4
BaP	0.9997	0.20	90.8	5.7	7.6	86.0	5.1	6.3	93.8	6.4	6.8
DahA	0.9994	0.20	92.7	7.1	8.2	90.6	6.3	7.4	95.2	3.9	5.2
IcdP	0.9987	0.20	74.6	10.4	11.8	71.3	9.5	10.7	77.6	8.7	9.5
BghiP	0.9996	0.20	78.5	6.9	5.8	75.1	7.3	8.5	83.6	5.6	7.1

### 2.6 Dietary exposure estimation

A common method to estimate daily intake for each PAH was the combination of contamination data with food consumption levels. Generally speaking, this estimation was calculated by an integration of mean levels of individual PAHs with the food consumption assumption of adult population with a body weight of 60 kg (Akpambang et al., 2009; Alomirah et al., 2011; Kao, Chen, Huang, Chen, & Chen, 2014). According to the data from the Chinese National Nutrition Survey in 2012, the average level of meat consumption was 89.7 g/day (He et al., 2016). Besides, a worst‐case scenario was estimated based on the maximum PAH contamination levels obtained from the samples analyzed.

### 2.7 Health risk estimation

Since individual PAHs have different ability to produce a toxic effect, the toxic equivalency factors (TEFs) (Table 3) are utilized for the estimation of the potential risk of PAH compounds (Essumang et al., 2013; Jiang et al., 2015; Li, Wu et al., 2016). BaP, the most potent carcinogenic and representative PAH, has a reference TEF value of 1. For a further step, in order to assess the hazard of PAH compounds, the toxicity equivalency quotient (TEQ), expressed as the BaP equivalent concentrations, was obtained by multiplying the concentration of each PAH with its TEF (Jiang et al., 2015; Li, Wu et al., 2016; Xia et al., 2010). The TEQ_BaP_ of food was calculated according to Equation (1).

**Table 3:** Summary of occurrence, mean contamination levels (μg/kg), and toxicity equivalency quotient concentrations (TEQ_BaP_, ng/kg) of individual PAHs in 52 grilled and fried meat products

PAHs	Occurrence/total (%)	Mean	TEFs	TEQB(a)p/(ng/kg)
Nap	52/52 (100.0)	23.0	0.001	23.0
Ace	51/52 (98.1)	7.99	0.001	7.99
Fle	48/52 (92.3)	7.31	0.001	7.31
Phe	48/52 (92.3)	8.21	0.001	8.21
Ant	43/52 (82.7)	1.77	0.01	17.7
Flu	45/52 (86.5)	6.75	0.001	6.75
Pyr	47/52 (90.4)	5.61	0.001	5.61
BaA	26/52 (50.0)	0.56	0.1	56
Chr	28/52 (53.8)	0.69	0.01	6.9
BbF	22/52 (42.3)	0.36	0.1	36
BkF	16/52 (30.8)	0.2	0.1	20
BaP	23/52 (44.2)	0.36	1	360
DahA	9/52 (17.3)	0.13	1	130
IcdP	13/52 (25.0)	0.21	0.1	21
BghiP	10/52 (19.2)	0.13	0.01	1.3

(1) $${\text{TEQ}}_{\mathrm{BaP}}=\times {\text{TEF}}_{i}$$

where C_*i*_ is the determined PAH value for the “ith” compound with the defined TEF_*i*_. The carcinogenic potencies of 15 PAHs were estimated as the sum of each individual TEQ_BaP_.

The incremental lifetime cancer risk (ILCR) of population groups in Shandong associated with dietary exposure of PAHs in meat was calculated by Equation (2) based on our and others’ reported methods (Jiang et al., 2015; Li, Wu et al., 2016):

(2) $$\text{ILCR}={\text{TEQ}}_{\mathrm{BaP}}\times \text{IR}\times \text{EF}\times \text{ED}\times \text{SF}\times \text{CF}/\left(\text{BW}\times \text{AT}\right)$$

where ILCR = the incremental lifetime cancer risk of dietary exposure; IR = the ingestion amount of meat products (0.0897 kg/day), which was obtained from the data of the Chinese National Nutrition Survey in 2012 (He et al., 2016). SF = the oral cancer slope factor of BaP, which obeys lognormal distribution with a geometric mean of 7.3 mg kg^−1^ day^−1^ (USEPA, 2001). ED = the exposure duration (year) (for children: ED = 7; for adolescents: ED = 7; for adults: ED = 43; for seniors: ED = 10) (Li, Dong et al., 2016; Xia et al., 2010). BW = average body weight during exposure duration (kg); AT = the average life span for carcinogens (equal to 76 years in China, 27,740 days, which was based on the World Health Statistics released by the World Health Organization (WHO) (Xu, Zhang, Yang, Zou, & Zhao, 2014). EF = the exposure frequency (365 days/year); CF = the conversion factor (10^−6^ mg/ng).

## 3 RESULTS AND DISCUSSION

### 3.1 Contamination levels of PAHs in meat samples

The developed and validated method was further utilized for the quantitative analysis of 15 PAHs in grilled and fired meat products. Table 3 displayed the occurrence and mean contamination level of each single PAH in all meat products. Briefly, the concentrations of total PAHs (Σ_15_PAH) in 52 different meat samples ranged from 8.23 to 341 μg/kg with a mean contamination level of 63.3 μg/kg. The proportions of the light PAHs (LPAHs, include Nap, Ace, Fle, Phe, Ant, Flu, Pyr, BaA, and Chr) above the LOQ were higher than the heavy PAHs (HPAHs, include BbF, BkF, BaP, DahA, BghiP, and IcdP). The trend was similar with those in edible oils in China, Italy, and Kuwait (Alomirah et al., 2010; Jiang et al., 2015; Moret et al., 2005). Concerning on the individual PAH, Nap was the dominant PAH with a mean level of 23.0 μg/kg, which was consistent with the results of smoked meats in southwest China (Li, Dong et al., 2016).

#### 3.1.1 Contamination levels of PAHs in grilled meat products

As shown in Table 4, the concentrations of Σ_15_PAH in 23 grilled meat samples were ranged from 12.0 to 341 μg/kg, indicating a wide variability in tested samples. The mean concentration was 80.0 μg/kg. The average levels of PAH8, PAH4, and BaP were 3.34, 2.65, and 0.33 μg/kg, respectively. Higher mean levels of the light PAHs (LPAHs, include Nap, Ace, Fle, Phe, Ant, Flu, Pyr, BaA, and Chr) than the heavy PAHs (HPAHs, include BbF, BkF, BaP, DahA, BghiP, and IcdP) were observed. Among the LPAHs, Nap was detected at the highest average levels (23.9 μg/kg), followed by Fle (11.4 μg/kg), Phe (11.1 μg/kg), and Ace (9.98 μg/kg), respectively. Among the HPAHs, BbF was the dominant analyte with a mean level of 0.43 μg/kg, followed by BaP, BkF (0.23 μg/kg), and IcdP (0.18 μg/kg), respectively.

**Table 4:** Median and mean concentrations (μg/kg) and range of individual PAHs and the sum of PAH8 and PAH4 in grilled and fried meat products

	Grilled meat products (*n* = 23)			Fried meat products (*n* = 29)		
	Median	Mean	Range	Median	Mean	Range
Nap	16.4	23.9	3.86–91.8	16.7	22.3	1.86–75.4
Ace	6.3	9.98	0.95–57.9	6.29	6.41	<RL^a^–18.9
Fle	4.62	11.4	<RL–92.8	3.23	4.09	<RL–13.7
Phe	6.46	11.1	<RL–80.3	5.84	5.89	<RL–15.7
Ant	0.78	2.69	<RL–16.3	0.52	1.05	<RL–4.55
Flu	4.18	8.35	<RL–73.0	1.32	5.49	<RL–71.9
Pyr	2.26	9.23	<RL–82.9	1.95	2.74	<RL–22.6
BaA	0.51	0.91	<RL–5.44	<RL	0.27	<RL–1.61
Chr	0.67	0.98	<RL–4.04	<RL	0.45	<RL–5.62
BbF	<RL	0.43	<RL–2.17	<RL	0.31	<RL–2.50
BkF	<RL	0.23	<RL–1.75	<RL	0.18	<RL–0.83
BaP	<RL	0.33	<RL–2.18	<RL	0.39	<RL–1.95
DahA	<RL	0.14	<RL–2.14	<RL	0.12	<RL–1.08
IcdP	<RL	0.18	<RL–1.34	<RL	0.24	<RL–1.68
BghiP	<RL	0.14	<RL–0.79	<RL	0.12	<RL–0.89
Σ_15_PAH	49.7	80.0	12.0–341	48.9	50.1	8.23–118
PAH8^b^	2.41	3.34	<RL–14.6	1.73	2.08	<RL–6.00
PAH4^c^	2.04	2.65	<RL–13.0	0.74	1.42	<RL–6.00

a Lower than the reporting limit of quantification.

b PAH8 includes the sum of BaA, Chr, BbF, BkF, BaP, DahA, BghiP, and IcdP.

c PAH4 includes the sum of BaA, Chr, BbF, and BaP.

The PAH levels observed here were compared with those reported for grilled meat products in published studies. Elhassaneen (2004) found that the range of 11 PAHs in charcoal‐broiled beef burgers varied from 0.31 to 14.95 μg/kg and the levels of the BaP concentrations were between 0.99 and 4.8 μg/kg. Reinik et al. (2007) determined the content of 12 PAHs in 14 Estonian home‐grilled meat products, finding the mean values of Σ_12_PAH ranged from 8.6 to 20 μg/kg. Farhadian, Jinap, Abas, and Sakar (2010) analyzed nine types of Malaysian popular grilled meat dishes and found that the total levels of Flu, BbF, and BaP ranged from 3.51 to 132 μg/kg. Alomirah et al. (2011) found that the total levels of 16 PAHs in whole grilled chicken, purchased from the State of Kuwait, ranged from 48.2 to 342 μg/kg with a mean concentration of 222 μg/kg.

Many factors may be responsible for the production of PAHs in meat foods resulting in a wide variability of the contamination levels. Generally speaking, PAHs were significantly formed from pyrolysis of organic matter during the meat grilling process at high temperature (Akpambang et al., 2009; Farhadian et al., 2010; Kao et al., 2014; Lee et al., 2016; Viegas, Novo, Pinto, Pinho, & Ferreira, 2012). Therefore, the grilling procedure and fat content in meat seem to be the major reasons for high PAH levels in meat products (Lee et al., 2016; Oz & Yuzer, 2016; Purcaro et al., 2013; Viegas et al., 2012). In briefly, fat drips from the meat samples onto the flames and subsequently burns resulting in smoke generation, which cause the formation of PAHs through the incomplete combustion of charcoal (Lee et al., 2016; Singh et al., 2016). In addition, some ingredients, especially those used in grilled step, may also contribute to the formation of PAHs (Farhadian et al., 2010).

#### 3.1.2 Contamination levels of PAHs in fried meat products

Correspondingly, the mean concentrations of Σ_15_PAH, PAH8, PAH4, and BaP (Table 4) in 29 fried meat samples were 50.1, 2.08, 1.42, and 0.39 μg/kg, respectively. In particular, in some samples, the total concentrations of PAH8, PAH4, and BaP were below the LOQ, which were similar with the trends in grilled samples. LPAHs were primary in all fried samples, and Nap, Ace, and Phe were the predominant ones, with the mean levels being 22.3, 6.41, and 5.89 μg/kg, respectively. Among the HPAHs, BaP was the highest one, followed by BbF (0.31 μg/kg) and IcdP (0.24 μg/kg). In short, the fried meat products were mainly suffered from LPAHs contamination, which was the same trend with the grilled meat products in this study.

Limit data on the contamination levels of PAHs in fried foods are available from the published studies. Perello, Marti‐Cid, Castell, Llobet, and Domingo (2009) reported that the total concentrations of 16 individual PAHs in fried meat and fish samples were ranged from 13.30 to 35.42 μg/kg. Olatunji, Fatoki, Opeolu, and Ximba (2015) found that the sum of BaP and BkF in the different fried fish samples was between 1.04 and 1.46 μg/kg. Recently, Li, Wu et al. (2016) reported that the sum concentrations of 16 PAHs and PAH4 in youtiao, a Chinese traditional fried food, were varied from 9.90 to 89.97 μg/kg and from 1.41 to 26.56 μg/kg, respectively. In general terms, the contamination of PAHs compounds found in fried foods was probably a consequence of high‐temperature processing and PAHs contamination of fried oils and raw materials (Oz & Yuzer, 2016; Rose et al., 2015). Moreover, the content variations of PAHs in fried foods depended on many factors, such as the fat content in food, penetration of oil, duration, temperature achieved, and air circulations (Li, Wu et al., 2016; Rose et al., 2015; Singh et al., 2016).

For a further step, the contamination levels of BaP and PAH4 in this study were compared with the MLs for them as defined in the EU Commission Regulation for smoked meat products. Among the analyzed samples, the concentrations of BaP ranged from <LOQ to 2.18 μg/kg with a mean level of 0.36 μg/kg. The incidence rate (23 out of 52 samples) was 44.2%, which was similar with the proportion (41%) reported by the EFSA for grilled and smoked meat samples from European countries (EFSA, 2008). For comparison, Alomirah et al. (2011) reported that BaP was found in 60% of the grilled and smoked foods in Kuwait, which was higher than the frequency reported here. Only one sample (2.18 μg/kg) exceeded the ML of 2 μg/kg for BaP, which was a grilled pork product. For PAH4, the mean concentration was 1.97 μg/kg, being significantly below the 12 μg/kg ML of the EU. Moreover, one sample (12.97 μg/kg) exceeded the ML, which was the same grilled pork product for BaP.

### 3.2 Dietary exposure estimation

Table 5 reported the estimated daily intake of individual PAHs, Σ_15_PAH, PAH8, and PAH4 by the average adult population from grilled and fried meat products. For grilled meat products, the average dietary intake of individual PAHs compounds ranged from 0.21 to 35.7 ng/kg bw/day, while the mean exposures calculated for Σ_15_PAH, PAH8, and PAH4 were 120, 4.99, and 3.96 ng/kg bw/day, respectively. When the worst‐case scenario was considered, the values for the sum of Σ_15_PAH, PAH8, and PAH4 raised to 510, 21.8, and 19.4 ng/kg^ ^bw/day, respectively. For fried meat samples, the average exposures of Σ_15_PAH, PAH8, and PAH4 were found to be 74.8, 3.11, and 2.12 ng/kg bw/day, whereas maximum intakes were estimated to be up to 176, 8.97, and 8.97 ng/kg bw/day, respectively. In addition, the mean daily intake of BaP for adult population from grilled and fried meat products was found to be 0.49 ng/kg bw/day (29.4 ng/day) and 0.58 ng/kg bw/day (34.8 ng/day), respectively.

**Table 5:** Estimated daily intake (ng/kg^ ^bw/day) of PAHs in grilled and fried meat products

	Grilled meat products (*n* = 23)		Fried meat products (*n* = 29)	
	Means^a^	Maximums^b^	Means	Maximums
Nap	35.7	137	33.4	113
Ace	14.9	86.6	9.58	28.3
Fle	17.0	139	6.11	20.5
Phe	16.7	121	8.81	23.5
Ant	4.02	24.4	1.57	6.80
Flu	12.5	109	8.21	107
Pyr	13.8	124	4.10	33.8
BaA	1.36	8.13	0.40	2.41
Chr	1.47	6.04	0.67	8.40
BbF	0.64	3.24	0.46	3.74
BkF	0.34	2.62	0.27	1.24
BaP	0.49	3.26	0.58	2.92
DahA	0.21	3.20	0.18	1.61
IcdP	0.27	2.00	0.36	2.51
BghiP	0.21	1.18	0.18	1.33
Σ_15_PAH	120	510	74.8	176
PAH8^c^	4.99	21.8	3.11	8.97
PAH4^d^	3.96	19.4	2.12	8.97

a The data for total samples.

b The data for the samples with the maximum contamination levels.

c PAH8 includes the sum of BaA, Chr, BbF, BkF, BaP, DahA, BghiP, and IcdP.

d PAH4 includes the sum of BaA, Chr, BbF, and BaP.

The estimated dietary intakes of PAH compounds are compared with the data obtained in published studies. Reinik et al. (2007) reported that the estimated mean daily intake of BaP by the Estonia population from the consumption of meat products was 0.45 ng/kg^ ^bw/day (29 ng/day). Akpambang et al. (2009) found that the dietary exposure to BaP and PAH8 from commercially smoked/grilled fish and meat products in Nigerian varied between 4.0 and 52.0 ng/kg bw/day, and between 58.1 and 269.8 ng/kg bw/day, respectively. Cirillo et al. (2010) reported that the daily median exposure values of BaP, PAH4, and PAH8 in primary school children were 5, 28, and 54 ng/kg bw/day, respectively. Rozentale et al. (2015) reported that the average consumer exposure to BaP and PAH4 from smoked meat products produced in Latvia was 0.33 and 2.91 ng/kg bw/day, respectively. Alomirah et al. (2011) evaluated the mean dietary intake to BaP and PAH8 for the adult Kuwait population from grilled and smoked foods was 9.20 and 95.7 ng/day, respectively. In particular, based on an average consumption across Europe (132 g/day), the EFSA (2008) reported the mean dietary intake of Bap and PAH8 from meat and meat products was 42 and 279 ng/day, respectively. Generally speaking, the degree of PAH dietary intake depends on both the nutritional habits of the local population and the contamination levels of PAHs in foods (Kao et al., 2014; Reinik et al., 2007). Therefore, the daily exposure of PAHs for the consumers, who frequently eat large quantities of meat products, might be considerably more than the average data.

### 3.3 Health risk estimation

According to USEPA (2001), the ILCR model was utilized for the health risk assessment of Shandong population caused by dietary PAHs exposure. Generally, additional human cancer risk of one in a million over a 70 years life span (ILCR = 10^−6^) is regarded as an acceptable or inconsequential level, while a one in a ten thousand chance (ILCR = 10^−4^) or greater is considered to be a serious level (Jiang et al., 2015; Kao et al., 2014; Xia et al., 2010). In the present study, the values of ILCR were estimated to 2.69 × 10^−6^, 1.14 × 10^−6^, 3.75 × 10^−6^, and 1.02 × 10^−6^, for children, adolescents, adults, and seniors in a 76‐year life span, respectively. Therefore, health risk assessment of dietary exposure to grilled and fried meat products was in the USEPA acceptable level, indicating a potential cancer risk potency. Among the four groups, adults suffered from highest carcinogenic risk, followed by children, adolescents, and seniors. The trend was similar with published data obtained in previous studies (Ding, Ni, & Zeng, 2013; Jiang et al., 2015; Li, Wu et al., 2016; Xia et al., 2010). In particular, the body weight of children was significantly lower than others, which caused a relatively high‐risk value for children. Therefore, it should be emphasized that children were the most sensitive group to PAHs exposure and special attention should be paid for their health (Ding et al., 2013; Marti‐Cid, Llobet, Castell, & Domingo, 2008).

A comparison with previous studies was given. Xia et al. (2010) reported that ILCR due to the dietary exposure of PAHs in Taiyuan ranged from 7.08 × 10^−6^ to 4.04 × 10^−5^ for different groups, which were higher than the results in this study. Duan et al. (2016) reported that the median value of estimated ILCR attributable to PAH dietary intake was 6.65 × 10^−5^, which was also higher than our present result. Li, Dong et al. (2016) demonstrated that the ILCR values due to PAHs exposure from intake of smoke meats were range from 4.46 × 10^−7^ to 4.64 × 10^−6^ for eight groups in southwest China. Kao et al. (2014) reported that the estimated ILCR value due to dietary PAHs exposure from kindling‐free charcoal‐grilled meat products was no more than 0.26 × 10^−6^, indicating a slight cancer risk potency. As discussed in published studies, the health risk assessment of PAHs exposure is a complex issue. The different ILCR values can be explained, in part, by the fact that the exposure duration, the PAH contamination levels, and daily food consumption amounts were different in these studies (Li, Wu et al., 2016). In short, a higher health risk assessment is usually associated with higher contamination and consumption levels.

Although the risk levels due to PAHs exposure for Shandong population were at acceptable range, it can be much higher for people who often eat large amounts of meat products. In particular, with rapid economic growth in the past three decades, a dramatically increasing trend of meat consumption has been observed. Moreover, other types of foods suffering with PAHs contamination were not taken into account in the present health risk estimation. If all PAHs exposure routes via food ingestion were included, the estimated cancer risk level for local population would be greater than the values obtained here. Therefore, with the aim to protect food safety and human health, it is still necessary to control processing conditions to minimize PAH contamination of commercial grilled and fried meat products.

## 4 CONCLUSIONS

In summary, the contamination levels of 15 PAHs in 52 grilled and fried meat products in Shandong Province, China, were determined by a sensitive HPLC method. Then, the obtained data were used to estimate the daily intake of individual PAHs by local population. Finally, the health risk estimation due to dietary PAHs exposure was successfully estimated. Hence, the present study was the first attempt to provide baseline information of potential health risk for dietary exposure of PAH‐containing grilled and fried meats, which could be useful for health management of the consumers in Shandong Province, China.

## CONFLICT OF INTERESTS

The authors declare that they do not have any potential sources of conflict of interest.

## ETHICAL STATEMENT

This study does not involve any human or animal testing.
